# Accuracy of pharmaceutical company licensing predictions: projected versus actual licensing dates

**DOI:** 10.1111/jphs.12132

**Published:** 2016-03-22

**Authors:** Lucy Doos, Derek Ward, Andrew Stevens, Claire Packer

**Affiliations:** ^1^ NIHR Horizon Scanning Research and Intelligence CentreInstitute of Applied Health ResearchUniversity of BirminghamBirminghamUK; ^2^ Institute of Applied Health ResearchUniversity of BirminghamBirminghamUK

**Keywords:** Early awareness and alert systems, horizon scanning, licensing, pharmaceutical, prediction

## Abstract

**Objectives:**

To determine the accuracy of pharmaceutical companies' predictions of drug licensing timeframes for their products in late stage clinical development.

**Methods:**

We compared predicted licensing dates provided to the National Institute for Health Research Horizon Scanning Research and Intelligence Centre by pharmaceutical companies against actual marketing authorisation application (MAA) and marketing authorisation (MA) dates published by the European Medicines Agency for drugs granted authorisation between 2009 and 2013.

**Key findings:**

One hundred and twenty‐three drugs met our inclusion criteria. About 78% were new drugs and 16% had orphan designation. Less than half (44%) and less than a quarter (24%) of MAA and MA predictions respectively were considered accurate (same month or 1 month either side of the actual date). Pharmaceutical companies were significantly more accurate in predicting MAA dates than MA dates (*P* < 0.001). For accurate predictions, the mean duration between the prediction being made and the actual MAA and MA dates were 17.5 and 18.7 months respectively. Out of the total 108 MA predictions, almost two‐thirds (65.4%, 16/26) of short‐term predictions (made in the 2 years prior to the actual MA) were accurate. For predicted dates that were earlier than the actual MA date, there was a positive relationship between accuracy and the time between the prediction and authorisation.

**Conclusions:**

Even in predicting near events from well‐informed sources, accuracy is imperfect. There appears to be an optimum time for the provision of accurate information on predicted MAA and MA dates for drugs. This information is crucial for effective early awareness and alert activities.

## Introduction

New drugs and new indications for existing licensed drugs have the potential to bring about important change in medical practice leading to benefits for patients, clinicians and health services. They can improve the quality of patients' lives through improved management of disease, enable patients to remain in their homes rather than in hospitals, simplify treatment schedules, and allow clinicians to treat patients more effectively and efficiently.^1^ They can, of course, also confer net harm if they displace more cost‐effective treatments. Before a drug can be marketed for a specific indication, it undergoes a process of licensing by the applicable medicines regulator, which then issues marketing authorisation (MA). The regulation of medicines ensures its safety and the protection of public health. In the UK, two regulators perform this function, the European Medicines Agency (EMA), which aims to streamline the licensing process and ensure a homogeneous regulatory policy throughout the European Union, and the UK Medicines and Healthcare Products Regulatory Agency.

Early identification of imminent technologies enables decision makers to plan further evaluation, plan future investment, decide on the allocation of resources, identify requirements for implementation such as staff training and the development of facilities, and make changes to treatment and management pathways.^2^ This, in turn, helps health systems incorporate such innovation in a sustainable way and facilitates appropriate adoption.^3^, ^4^, ^5^, ^6^, ^7^ Timely evaluation and planning requires accurate information about probable launch dates, and a lack of accurate intelligence can hinder informed decision making with undesirable health and financial consequences.^8^ Most pharmaceutical companies begin speculation about the eventual date of licensing during phase II clinical development.^9^ These discussions continue through the rest of the development process until the application for regulatory approval.

Many countries have established early awareness and alert (EAA) systems (also known as horizon scanning or early warning systems) to provide decision makers with information on new health technologies prior to their introduction and adoption into health systems.^7^, ^10^ The National Institute for Health Research Horizon Scanning Research and Intelligence Centre (NIHR HSRIC) 11 in England is an EAA system that provides advance notice on new and emerging health technologies and interventions, including drugs that are likely to have a significant impact on the English National Health Service and/or patients within the next 2–3 years.^12^ The system informs the topic selection and timing of health technology assessments (appraisals) undertaken by the National Institute for Health and Care Excellence (NICE). Key features of the NIHR HSRIC methods include extensive and proactive contact with pharmaceutical companies to identify products in development and obtain company predictions for future dates of marketing authorisation application (MAA or ‘filing’) and marketing authorisation (MA or ‘licence’) with the EMA. Scanning for new medicines also includes scrutiny of relevant commercial and general media, scientific publications, commercial R&D databases and access to the *UK PharmaScan* database of pharmaceuticals in development.^13^ The NIHR HSRIC aims to produce information on new drugs and new indications for existing licensed drugs around 20 months and around 15 months prior to launch respectively.^14^

Prediction, however, is not always accurate, and depends on the availability and quality of data. It is generally assumed that as a technology nears licensing, any predictions made about the timing of regulatory approval will be increasingly accurate.^15^ Predictions from pharmaceutical companies on the anticipated timing of MAA and MA are of crucial importance to the work of the NIHR HSRIC. We aimed to determine how accurate such predictions have been, and if they varied according to the whether this was the first or subsequent indication for the drug, by orphan designation, as well as the time from making the prediction to subsequent licensing.

## Methods

### Design

A cross‐sectional study comparing predicted MAA and MA dates obtained from NIHR HSRIC contacts with pharmaceutical companies against actual MAA and MA dates for both new drugs and new indications for existing licensed drugs awarded MA between 2009 and 2013 (inclusive).

### Data sources

Information on drugs licensed between 2009 and 2013 (inclusive, and including those subsequently withdrawn), their indication, orphan designation, and dates of MA and MAA were obtained from the EMA website.^16^

Data on individual predicted MAA and/or MA dates were obtained from the NIHR HSRIC's confidential information system, which is populated with data obtained directly from commercial pharmaceutical companies. Data on whether the MA represented a new drug or a new indication for an existing licensed drug were obtained from the EMA website 16 and relevant editions of the British National Formulary.^17^

While all new drugs (new chemical entities and new biologic products) and new indications for existing licensed drugs receiving MA between 2009 and 2013 were eligible for inclusion in this study, only those with a company prediction for anticipated future MAA or MA dates available on the NIHR HSRIC information system were included in the analysis. Generic drugs, biosimilars and blood products were excluded, as were vaccines and diagnostic agents, which have a different assessment and market access pathway in the United Kingdom.

### Data handling and analysis

Differences in the duration between the predicted and actual MAA and MA dates were calculated to the nearest month. A prediction was considered accurate when the actual MAA or MA date fell in the same month as the prediction or in the month before or after the prediction.

Statistical analysis was carried out using IBM SPSS statistics (version 21) for Windows. Descriptive analyses were presented as means and standard deviations (SD) for normally distributed continuous variables, medians for skewed continuous data and percentages for dichotomous variables. Significant differences were determined using ANOVA for continuous normally distributed data and χ^2^ for dichotomous variables.

## Results

### Data availability

One hundred and ninety‐four new drugs and new indications for existing licensed drugs were awarded MA by the EMA in the 5‐year study period between 2009 and 2013 (inclusive), of which 123 (63.4%) had a company prediction of the likely MAA and/or MA date recorded in the NIHR HSRIC information system. Two‐thirds of these 123 drugs (65%) had both predicted MAA and MA dates available on the NIHR HSRIC database. More than three quarters of the drugs included in the analysis were new drugs (78.3%) rather than new indications for existing licensed drugs, and the majority did not have an orphan designation (84.0%).

### Accuracy of company predictions for MAA and MA dates

Less than half (43.8%) and less than a quarter (24.1%) of MAA and MA predictions, respectively, were regarded as accurate. Of the 80 drugs where predictions were available for both MA and MAA, only 9 (11.3%) drugs had accurate predictions for both MAA and MA (Table 1). The majority of errors were optimistic ones; with 28.8% of those predictions were expected to happen before the actual MA/MAA dates. The differences between the accuracy of predictions were statistically significant (McNemar–Bowker test = 14.4, df = 3, *P* = 0.002). Company predictions for MAA dates ranged from 72 months before the actual MAA date to 28 months after the actual MAA date. Company predictions for MA dates ranged from 75 months before the actual MA date to 15 months after the actual MA date.

**Table 1:** Pharmaceutical company predictions for MAA and MA compared to the actual MAA and MA dates for drugs awarded MA between 2009 and 2013^a^

Accuracy of prediction MA prediction	Accuracy of prediction MAA prediction			
	Accurate prediction	Predictions earlier than the actual date	Predictions later than the actual date	Total
Accurate prediction	9 (11.3)	4 (5.0)	5 (6.3)	18 (22.5)
Predictions earlier than the actual date	20 (25.0)	23 (28.8)	0 (0.0)	43 (53.8)
Predictions later than the actual date	8 (10.0)	3 (3.8)	8 (10.0)	19 (23.8)
Total	37 (46.3)	30 (37.5)	13 (16.3)	80 (100.0)

Values within parentheses are expressed as percentage. MAA, marketing authorisation application; MA, marketing authorisation.

a McNemar–Browker test = 14.36, df = 3, *P* = 0.002.

There was no difference in the percentage accuracy of MAA predictions between new drugs (44.3%) and new indications (45.5%). However, for MA, only 24.0% of predictions for new drugs were accurate compared to 30.8% for new indications, but this difference was not statistically significant. For drugs with predictions earlier than the actual dates, the mean number of months difference from the actual dates was less for new drugs (8.6 and 10.4 for MAA and MA respectively) than for new indications (14.6 and 7.3 months for MAA and MA respectively). For drugs with predictions later than the actual dates, the mean difference between actual and predicted dates was greater for new drugs (6.4 months for MAA and 4.8 for MA) than for new indications (3.0 months for MAA and 3.0 for MA).

Orphan designation made little difference to the percentage of accurate predictions (MAA, 50% and 43.2% and MA, 23.8% and 25%, for those with and without orphan designations respectively), though an increase in the spread of data was observed in the accuracy of prediction for drugs without orphan designations. However, for drugs with predictions earlier than the actual dates, the mean difference between actual and predicted dates was less for drugs with an orphan designation (4.3 for MAA and 8.8 for MA) than those without an orphan designation (10.7 for MAA and 10.3 for MA), but these differences were not statistically significant. However, for drugs with predictions later than the actual dates, this pattern was observed only for MAA (mean difference between actual and predicted dates for MAA, 4.3 for orphan and 10.7 for non‐orphan drugs; mean difference between actual and predicted dates for MA, 7.3 for orphan and 4.2 for non‐orphan drugs).

### Length of time between the prediction being received, actual MAA/MA date and prediction accuracy

More than half of company predictions for MA dates available on the NIHR HSRIC database (58.3%) were received within 2 years of the actual MA date (Table 2), and almost two‐thirds of accurate predictions (65.4%, 17 of 26) were found in this group. For predictions received more than 24 months prior to final MA, only 20% (9 of 45) was accurate and the vast majority was optimistic (67%, 30 of 45).

**Table 2:** Length of time between receiving predictions and actual MA date, and the accuracy of prediction

Accuracy of prediction	Length of time between receiving the prediction and MA date					
	0–6 months	7–12 months	13–18 months	19–24 months	More than 24 months	Total
Accurate prediction	3 (11.5)	3 (11.5)	7 (26.9)	4 (15.4)	9 (34.6)	26 (100)
Predictions earlier than the actual date	3 (5.1)	6 (10.2)	13 (22.0)	7 (11.9)	30 (50.8)	59 (100)
Predictions later than the actual date	1 (4.3)	3 (13.0)	8 (34.8)	5 (21.7)	6 (26.1)	23 (100)
Total	7 (6.5)	12 (11.1)	28 (25.9)	16 (14.8)	45 (41.7)	108 (100)

Values are expressed as *n* (%). MA, marketing authorisation.

Cases where the company prediction was accurate for MAA had a statistically significantly shorter median length of duration between the date when predictions was received and the actual MAA date (Table 3, median 17.0 months, *P* < 0.0001). For the nine drugs where the predictions were accurate for both MAA and MA, the mean duration between the predictions was received and the actual MA date was 15.8 months (SD 6.04, median 17, minimum 4 and maximum 26).

**Table 3:** Median difference in duration between the date of receiving prediction and the actual MA date, and accuracy of company prediction for MA and MAA

Accuracy of prediction (number for MAA, MA)	Median difference in duration in months	
	MAA	MA
Accurate prediction (39, 26)	17.0	18.5
Prediction date earlier than the actual date (33, 59)	25.0	25.0
Prediction date later than the actual date (14, 23)	19.0	18.0
*P*‐value (Kurskal–Wallis Test)	<0.0001	<0.0001

MAA, marketing authorisation application; MA, marketing authorisation.

**Figure 1:**
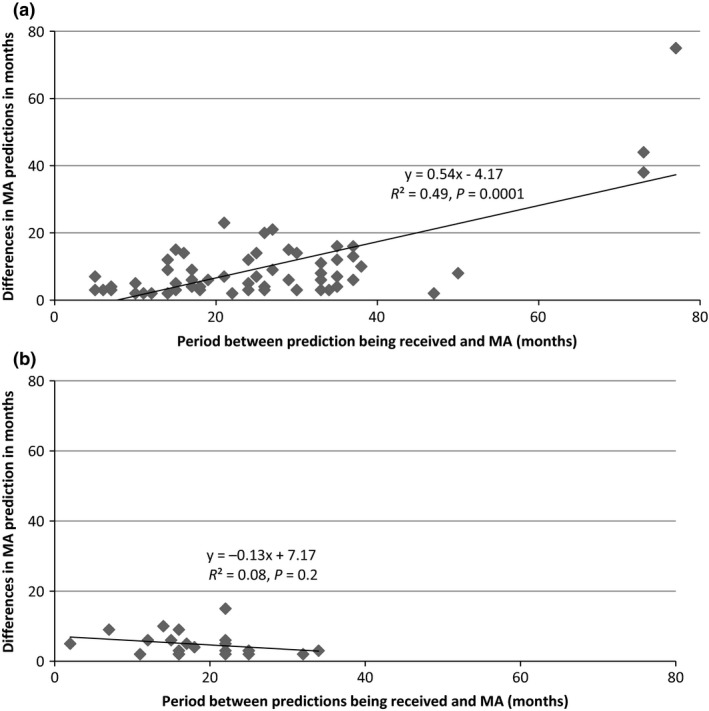
Time period between receiving predictions and actual MA dates compared to the difference between the company predicted and actual MA dates for predictions earlier (a) and later (b) than the actual MA date.

For company predictions that were earlier than the actual MA date, there was a positive relationship between the length of time between making the prediction and the actual MA date, and the accuracy of prediction (*y* = 0.54*x*−4.17, *r* = 0.70, *R*² = 0.49, *P* = 0.0001, Figure 1a). Visual inspection of the scatter plot suggested that the three drugs with predictions received more than 5 years before actual MA date may be outliers. After their exclusion, there still remained a positive, but much weaker relationship between the length of time between receiving the company prediction and the actual licensing date, and the accuracy of prediction (*y* = 0.13*x*−4.58, *r* = 0.26, *P* = 0.05). In contrast, no statistically significant relationship between prediction accuracy and the length of time between the prediction being received and actual MA date was seen for company predictions that were subsequently found to be later than the actual MA date (Figure 1b).

## Discussion

### Major findings of this study

Our analysis showed that most company predictions of licensing dates were inaccurate, with less than half of pharmaceutical company predictions for MAA timing and less than a quarter of their predictions for MA timing falling within our definition of accurate (the same month or the month either side of the actual date). More than half of company predictions for MA were optimistic, being earlier than the actual date. Our findings regarding the accuracy of pharmaceutical company predictions agree with the general comments of other authors, who suggest that one cannot expect fully accurate predictions and that by their very nature some predictions will inevitably be wrong.^18^, ^19^, ^20^ Accordingly, there are inherent limits to the accuracy of healthcare horizon scanning programmes and the future will always be uncertain.

Pharmaceutical companies were more accurate in predicting the dates for MAA than for MA, which may be explained by the date of filing being at least partly under the company's control, while the final MA date is primarily determined by the regulator. The maximum time taken for the EMA to conduct its review is limited by legislation, so that variations in the time taken for drugs to be awarded authorisation depends on whether more information is requested from the company to meet regulatory requirements. In this study, neither orphan designation nor drug development status (in terms of being a new drug or a new indication for an existing licensed drug) made a significant difference to the accuracy of company predictions.

### Implications for EAA systems

Estimation of the potential timing of approval for new and emerging drugs is crucially important for early decision making and appropriate planning. A major objective of an effective EAA system is to provide sufficient notice to policy makers before a new drug or technology diffuses into a healthcare system, and monitoring of drug licensing is critical with accuracy a fundamental criterion.^21^, ^22^ Acknowledging the challenge, the NIHR HSRIC performs extensive and proactive contact with pharmaceutical companies to identify products in company development pipelines and obtain predictions for licensing dates.^12^

Making predictions is a complex process, and their accuracy and certainty is always open to question.^6^, ^23^ Perhaps unsurprisingly, predictions received nearer to the time were more likely to be accurate. This finding needs to be considered when designing effective EAA systems as increasing uncertainty must be recognised when predictions are to be received a long time before the actual event. Our results suggest that providing information on emerging drugs more than 2 years from estimated MAA or MA dates is likely to result in less accurate predictions.

### Strengths and limitations

This is the first study to look comprehensively at the accuracy of pharmaceutical companies' predictions for the expected dates of their drugs' MA application and subsequent authorisation. Although we have been able to use the NIHR HSRIC extensive internal information system to provide company predictions, we were limited by data availability: not all the drugs licensed in the period of our study had company predictions available on the NIHR HSRIC database, and not all drugs for which there were predictions had predicted dates available for both MAA and MA. In addition, the availability of the data depends on the NIHR HSRIC data collecting efforts. For this study, the last available information from the company prior to the NIHR HSRIC producing an output was taken as the final predicted MAA or MA date.

## Conclusion

This study suggests that the current timescales used by the NIHR HSRIC to inform the NICE topic selection process are valid and provide a reasonable balance between earliness and accuracy. But making predictions for drug licensing timeframes even when using well‐informed sources represents a challenge for effective EAA systems and the results presented here demonstrate the inherent difficulties.

## Declarations

### Conflict of interest

The authors do not report any conflicts of interest.

### Funding

This work is supported by the United Kingdom's National Institute for Health Research (NIHR).

### Authors' contribution

DW, AS and CP had the initial idea for the study, and it was designed by LD and DW. LD collected and analysed the data, while all authors were involved in interpreting the results. LD drafted the manuscript, which was circulated to all authors for critical revision. All authors had full access to the data and agreed the final draft of the manuscript for submission.
